# The genomic distribution map of human papillomavirus in Western China

**DOI:** 10.1017/S0950268821001175

**Published:** 2021-05-18

**Authors:** Ling Chen, Yan Dong, Jiao Li, Jinqiu Zhao, Dan Wang, Li Xu, Yue Wu, Huandong Liu, Jungao Lu, Zuoyi Yao, Xiaosong Li

**Affiliations:** 1 The Center of Experimental Teaching Management, Chongqing Medical University, Chongqing 401331, China; 2 Department of Microbiology and Immunology, Kunming Medical University, Kunming 650031, China; 3 Department of Obstetrics and Gynecology, The First Affiliated Hospital of Xi'an Jiaotong University, Xian, China; 4 Department of Infectious Diseases, The First Affiliated Hospital of Chongqing Medical University, Chongqing 400016, China; 5 Department of Clinical Laboratory, People's Hospital of Rongchang District, Rongchang, Chongqing 402460, China; 6 Department of Clinical Laboratory, the First Affiliated Hospital of AMU, Chongqing 400038, China; 7 Oncology Department, The First Affiliated Hospital of Chongqing Medical University, Chongqing 400016, China; 8 Department of Neurosurgery, People's Hospital of Tibet Autonomous Region, Lhasa 850000, China; 9 Department of Clinical Laboratory, The Third Affiliated Hospital of Guizhou Medical University, Duyun 558000, China; 10 Department of General Surgery, The Chengdu Fifth People's Hospital, Chengdu 611130, China; 11 Clinical Molecular Medicine Testing Center, The First Affiliated Hospital of Chongqing Medical University, Chongqing 400016, China

**Keywords:** Cervical cancer, China, genotypes, Human papillomavirus (HPV), prevalence

## Abstract

Human papillomavirus (HPV) has been confirmed as the causative agent for cervical cancer. In this study, a total of 301 880 women were recruited from four different regions of Western China, with 301 880 exfoliated cervical cell samples collected from women for DNA isolation and purification. The HPV genotype was tested by polymerase chain reaction. The overall HPV prevalence rate, high-risk (HR) HPV infection rate, low-risk (LR) HPV infection rate and mixed HPV infection rate was 18.24%, 79.14%, 12.56% and 8.30%, respectively. The four most common HR HPV subtypes were HPV-52, 16, 58 and 53, which accounted for 20.49%, 19.93%, 14.54% and 10.01%, respectively. In LR HPV genotype, HPV-6 ranked the highest (28.17%), followed by HPV-81 (9.09%) and HPV-11 (3.78%). HPV genotype subgroup analysis also showed that single-type infection was the most common (77.26%) among HPV-positive individuals. Among multi-infection genotypes, double infection was the most common with frequencies of 76.04%. The overall prevalence of HPV is high in Western China, whose distribution demonstrates different patterns across different ages and regions. Viral genotypes HPV 53, 6 were frequently detected in this population, which is worth of significant clinical attention.

## Introduction

Cervical cancer, a leading genital cancer, is considered as the third most common gynaecologic malignancy and the fourth most common cause of death from cancer in women, with an estimation of 570 000 new cases and 311 000 new deaths in 2018 (GLOBOCAN, 2018) [1, 2]. Compared with developed countries, the age-standardised incidence rate of cervical cancer is higher in developing countries (16.7 per 100 000 *vs.* 12.7 per 100 000 women-years, respectively) [3]. China accounts for around 14% of the world's annual cases of cervical cancer [4, 5]. Thus, cervical cancer remains a relatively heavy burden of public hygiene management with increasing morbidity and mortality rates of cervical cancer in young women in China [6, 7].

Human papillomavirus (HPV), a sexually transmitted DNA virus from the Papovaviridae family, has been confirmed as the causative agent for cervical cancer [8]. It is estimated that most sexually active adults have been infected by at least one HPV genotype [9]. If the infection with the high-risk (HR) HPV strains persists, which could be a well-established cause of cervical cancer [10]. More than 200 distinct HPV genotypes have been discovered to date, of which approximately 40 infect the mucosal epithelium of the anus and genital tract [11]. Generally, they are classified as HR HPV (carcinogenic HPV types, HR-HPV), low-risk HPV (non-carcinogenic HPV types, LR-HPV) and intermediate-risk HPV (IR-HPV) based on their carcinogenic risk or reported potential pathogenicity [12].

It is well known that this type of malignancy is one of the most preventable cancers. Currently, part of comprehensive strategies aimed at control of cervical cancer is based on vaccination against HPV and HPV-based screening programmes, which have been demonstrated to effectively eliminate the burden of cervical cancer worldwide [13, 14]. Nonetheless, the prevalence and genotype distribution of HPV infections are heterogeneous widespread (differences vary among nations and regions, as well as within a country), which resulted in progress towards prevention often frustrating [15]. Hence, an accurate understanding of the regional distribution characteristics of HPV genotypes is extremely important for both prophylactic vaccine-based HPV development and for HPV-based cervical cancer screening strategies.

Reports about large-scale data on the genotypic spectrum of HPV infection are limited in China. Therefore, we conducted a retrospective summary of the enormous amount of HPV genotypes distribution data in China, the overall prevalence, age-specific prevalence and genotype distribution of HPV in different regions were also calculated and analysed. This study would provide guidance for the development of future screening and prevention programmes.

## Materials and methods

### Ethical considerations, study population and sample collection

This investigation was approved (No. 2020-173) by the Ethics Committee of the First Affiliated Hospital of Chongqing Medical University Ethics Review Board and informed consents were obtained from all participants for inclusion in the study. Briefly, this retrospective study collected 301 880 samples from over eight clinical hospitals, women's health centres, clinics and physical examination centres located in four different provinces of China. For each woman, the HPV genotyping results and relevant clinical information, including age and regional data were all collected.

### DNA extraction and HPV genotyping

Exfoliated cervical cell samples were collected from women by gynaecologists or obstetricians using a specialised cervical sampler brush for DNA isolation and purification. Based on rapid flow-through hybridisation of nucleic acid molecules, a gene chip detection system for nucleic acids identification of 21 HPV types was provided by Kaipu Biochemical Company in Chaozhou, Guangdong, China. Among 21 HPV types, there were 14 HR-HPV types (16, 18, 31, 33, 35, 39, 45, 51, 52, 56, 58, 59, 66 and 68), six LR-HPV types (6, 11, 42, 43, 44 and 81) and one IR-HPV type (53).

Subsequently, all HPV tests were performed with an HPV genotyping panel (polymerase chain reaction (PCR)-reverse dot-blot hybridisation method). Briefly, according to manufacturer's instructions, PCR was performed in a 25-μl reaction mixture containing 1-μl extracted DNA, 0.75-μl DNA Taq polymerase and 23.25-μl PCR-mix solution containing primer system. The PCR cycling parameters were as follows: an initial step at 95°C for 9 min, and followed by 40 amplification cycles (denaturation at 95°C for 20 s, annealing at 55°C for 30 s, 72°C for 30 s and a final extension at 72°C for 5 min). After amplification, HPV genotyping was performed by hybridisation and RDB on the strips fixed with HPV type-specific probes. The HPV type-specific probes immobilised on nylon membranes were used for reverse-blot hybridisation and detection of all HPV genotypes in a single assay in accordance with the manufacturer's instructions. Simultaneously, in order to validate the HPV test, sterile water was used as the negative control, and specimens with known HPV genotypes as the positive control.

### Statistical analysis

Data were analysed with IBM SPSS version 21.0. Sample characteristics including age (women with unknown ages were excluded), region, HPV infection result and genotypes distribution characters were summarised using frequency distributions to generate the numbers and percentages. The *χ*^2^ test was adopted to compare the HPV prevalence or proportions among different groups. A two-sided *P*-value of less than 0.05 (*P* < 0.05) was considered statistically significant.

## Results

### Overall HPV infection prevalence and genotype distribution

A total of 301 880 samples were collected and detected by HPV genotype. The genotype test showed 55 071 samples were HPV positive, the overall HPV prevalence rate was 18.24%, of which 28.14% in Tibet Autonomous Region, 18.59% in Chongqing Municipality, 10.33% in Guizhou Province and 29.09% in Shaanxi Province. Among the four different provinces, autonomous regions and municipalities, there were significant differences in the HPV prevalence (*χ*^2^ = 6120.54, *P* < 0.001). The details of genotypes distribution were shown, respectively, in the following contents.

### Age and genotype distribution of HPV infection in different regions

#### The HPV prevalence in Tibet Autonomous Region

**Fig. 1.**
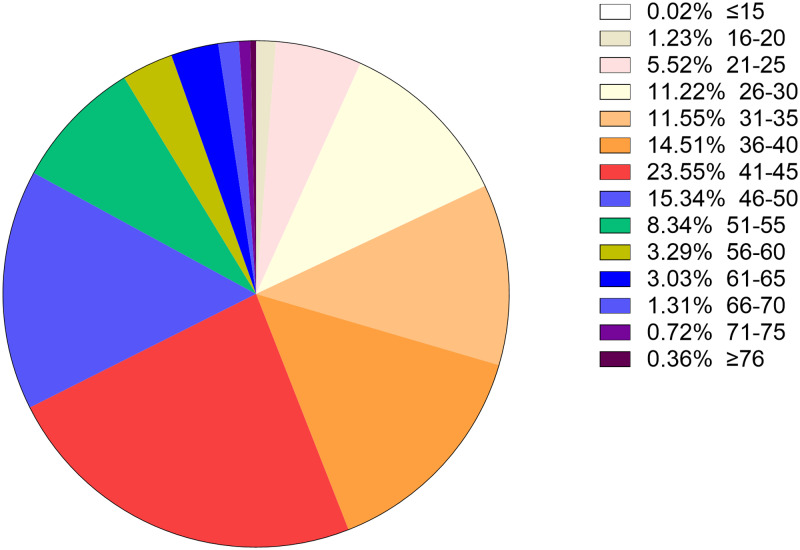
Age distribution of HPV infection in Tibet Autonomous Region.

**Fig. 2.**
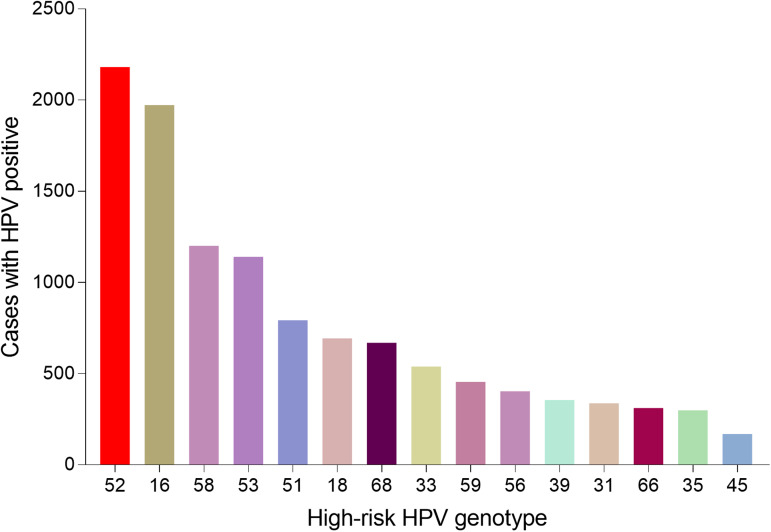
Genotype distribution of HR-HPV infection in Tibet Autonomous Region.

**Fig. 3.**
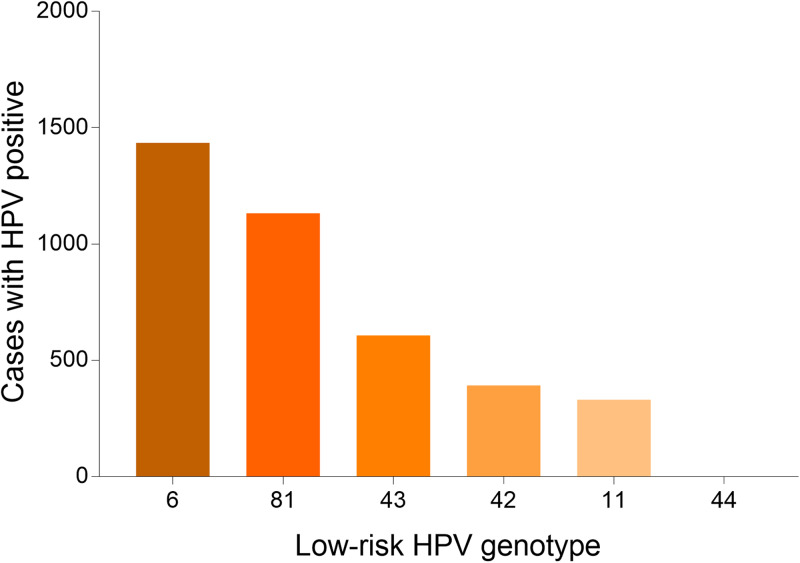
Genotype distribution of LR HPV infection in Tibet Autonomous Region.

A total of 36 073 samples were obtained in Tibet Autonomous Region and the HPV prevalence was identified as 28.14% (*n* = 10 150). The highest HPV detection rate was found in the group aged 36–50 years old (detection rate = 53.40%) (Fig. 1). Among HPV-positive women, the HR-HPV infection accounted for 73.51% (*n* = 7461). In HR-HPV, type 16 (19.44%) and 52 (21.50%) were the most prevalent genotypes, followed by 53 (11.23%) and 58 (11.83%). Among 1428 cases of LR-HPV infection, the two most common LR-HPV were type 6 (14.13%) and 81 (11.15%). The rates of single and multiple (infection with ⩾2 different HPV genotypes) infections were 70.35% (*n* = 7141) and 29.65% (*n* = 3009), respectively, with double infections rate at 71.32% among the multiple infections. Among the mixed (co-infection with HR-HPV and LR-HPV) infection cases, double HPV genotype infection took up over a half (*n* = 761). In multiple infection of HR-HPV, the double HPV genotype infection was also the highest in 1298 cases. The detailed genotype distribution of HPV infection was described in Figures 2 and 3. Overall, the prevalence of HPV infection in the Tibet Autonomous Region was dominated by single HR-HPV infection, especially type 16 or 52.

#### HPV infection in Chongqing municipality

**Fig. 4.**
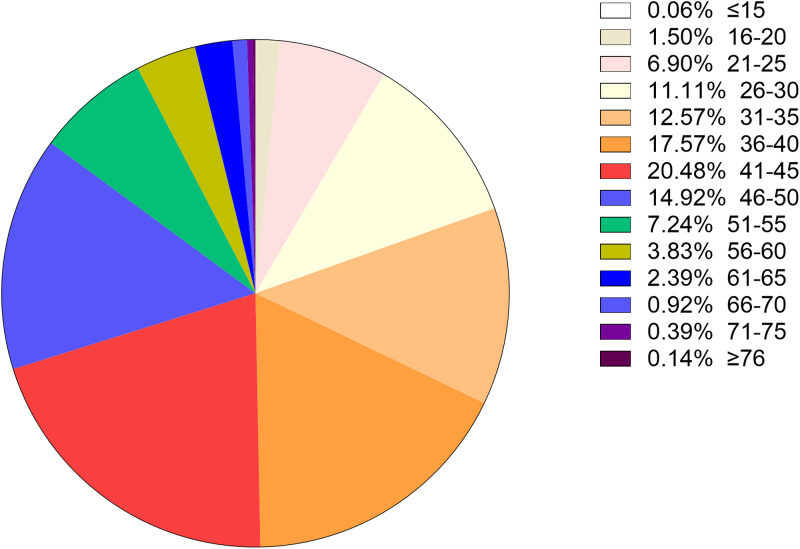
Age distribution of HPV infection in Chongqing Municipality.

**Fig. 5.**
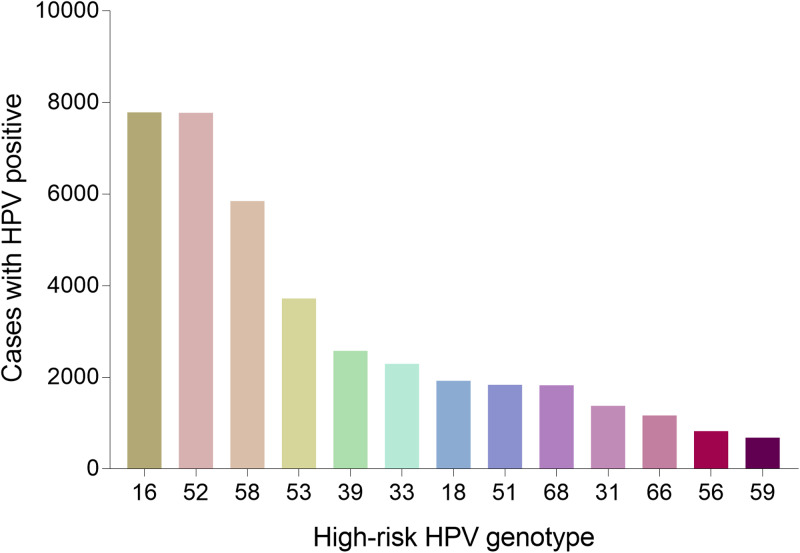
Genotype distribution of HR-HPV infection in Chongqing Municipality.

**Fig. 6.**
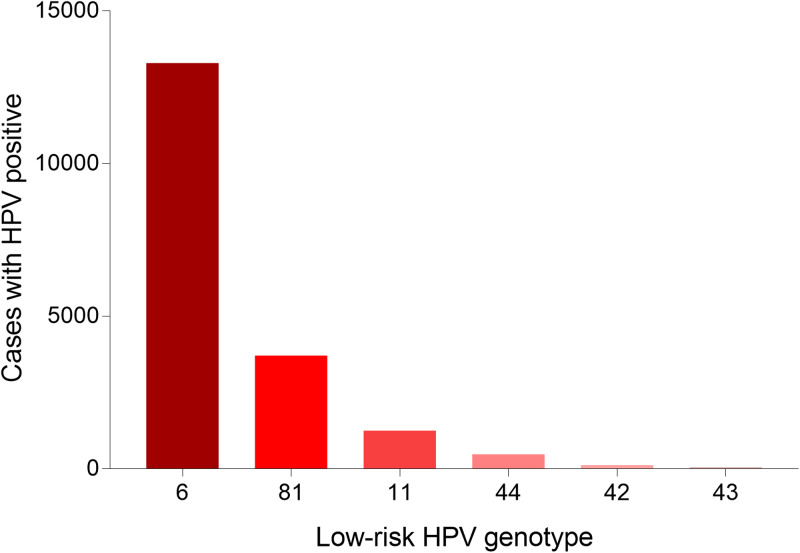
Genotype distribution of LR HPV infection in Chongqing Municipality.

A total of 37 389 women was detected as HPV positive among the 201 089 samples in Chongqing Municipality, with an HPV infection rate of 18.59%. Similar to Tibet Autonomous Region, the highest HPV detection rate was found in the group aged 36–50 years old (detection rate = 52.96%) (Fig. 4). Among the infected women, HR-HPV infection made up 81.32% (*n* = 30 406). Among HR-HPV infection, the three most prevalent types were type 16, 52 and 58, with frequencies of 20.82%, 20.80% and 15.64%, respectively. The most common LR-HPV types were HPV 6 (35.65%), even exceeded the most prevalent HR-HPV genotype (Figs 5 and 6). The rates of single and multiple infections were 79.00% (*n* = 29 537) and 21.00% (*n* = 7852), respectively, with a double infections rate at 77.06% among the multiple infections.

#### HPV infection in Guizhou Province

**Fig. 7.**
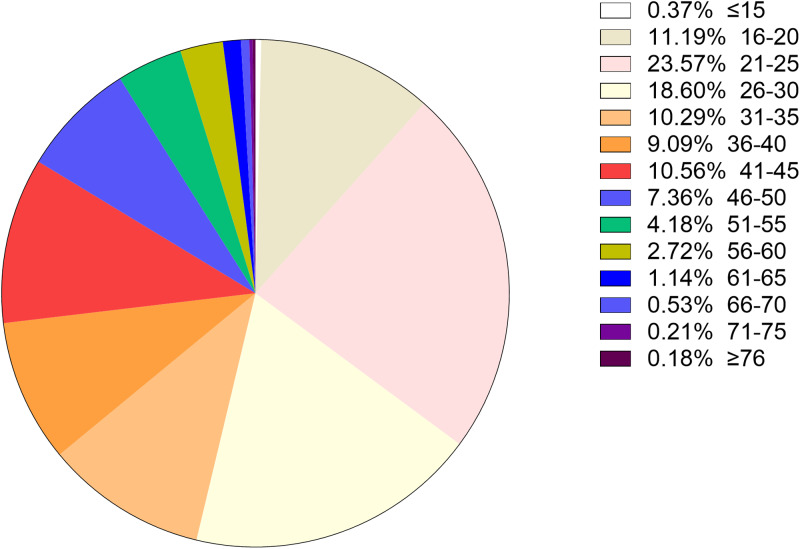
Age distribution of HPV infection in Guizhou Province.

**Fig. 8.**
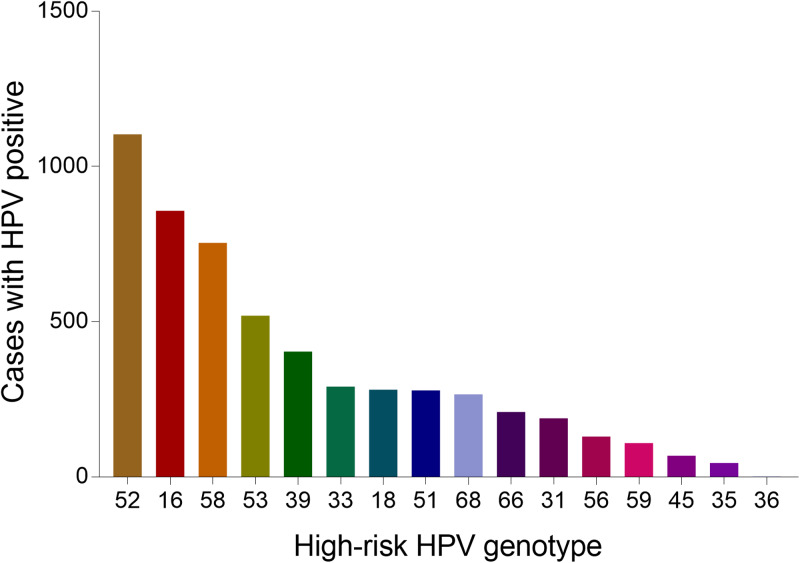
Genotype distribution of HR-HPV infection in Guizhou Province.

**Fig. 9.**
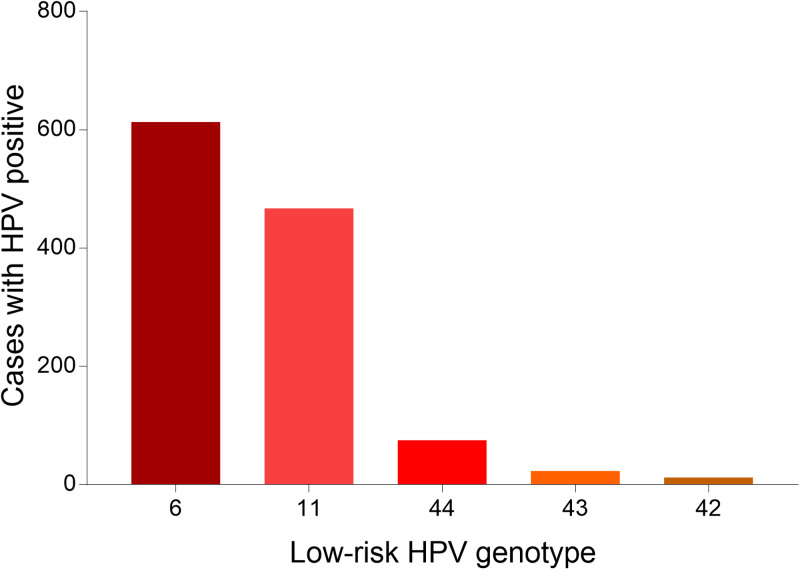
Genotype distribution of LR HPV infection in Guizhou Province.

Among 60 205 women of Guizhou Province, the prevalence of HPV infection was 10.33%, with 6219 cases identified as HPV positive. In all detected age groups, the highest HPV detection rate was found in the group aged 20–30 years old (detection rate = 43.18%) in Guizhou Province (Fig. 7). Among HPV-positive women, the HR-HPV infection took up 62.58% (*n* = 3892). Among HR-HPV infection, the four most prevalent types were type 52, 16, 58 and 53 in descending order. The most common LR-HPV types were HPV 6 and 11 (Figs 8 and 9). The rates of single and multiple infections were 79.71% (*n* = 4957) and 20.29% (*n* = 1262), respectively, with a double infections rate at 82.96% (*n* = 1047) among the multiple. The HR-HPV infection was also the dominant one in Guizhou Province with 3892 cases (62.58%).

#### HPV infection in Shaanxi Province

**Fig. 10.**
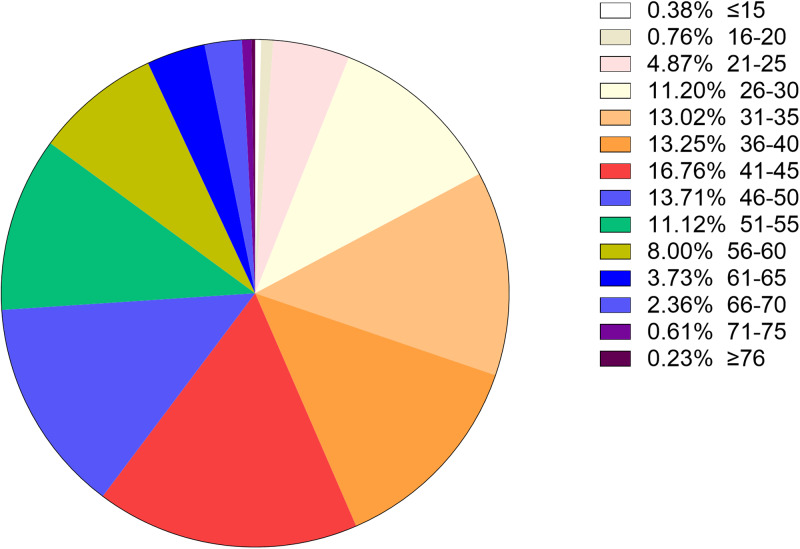
Age distribution of HPV infection in Shaanxi Province.

**Fig. 11.**
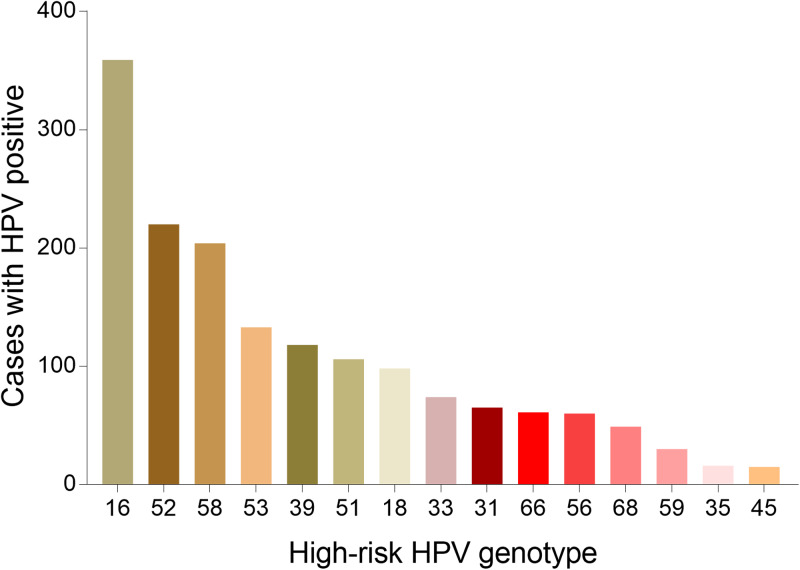
Genotype distribution of HR-HPV infection in Shaanxi Province.

**Fig. 12.**
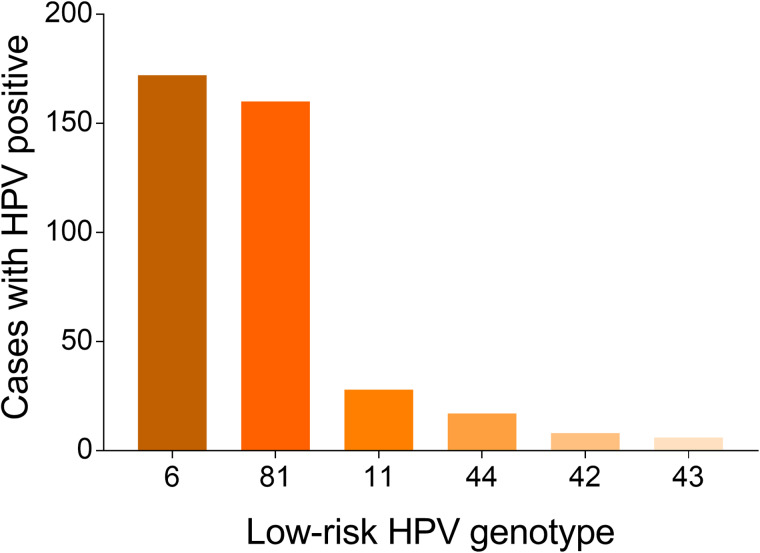
Genotype distribution of LR HPV infection in Shaanxi Province.

A total of 4513 samples was recorded in Shaanxi Province, with 1313 samples identified as HPV positive (29.09%). In all detected age groups, the highest HPV detection rate was found in the group aged 26–55 years old (detection rate = 79.06%) in Shaanxi Province (Fig. 10). Among HPV-positive women, the HR-HPV infection accounted for 80.05% (*n* = 1051). The four most prevalent HR-HPV types were type 16, 52, 58, 53 in descending order. The most common LR-HPV types were HPV 6 and 81 (Figs 11 and 12). The rates of single and multiple infections were 69.92% (*n* = 918) and 30.08% (*n* = 395), respectively. Among the multiple infections, double genotypes infections were the dominant one, which made up 69.62% (*n* = 275).

#### HPV genotype distribution in all positive samples

A total of 55 071 positive samples was detected in this study. We also analysed the genotype distribution for better decision making on the HPV vaccine tragedy. Notably, HPV 52 (11282, 20.49%), 16 (10974, 19.93%), 58 (8007, 14.54%), 53 (5513, 10.01%) were the top four prevalent genotypes among HR-HPV, and HPV 6 (15515, 28.17%), 81 (5008, 9.09%), 11 (2082, 3.78%) were the top three prevalent genotypes among LR-HPV. Table 1 showed the detailed genotype distribution.

**Table 1.** Genotype distribution of the total population

	Genotype	Cases	Prevalence (%)
HR-HPV	16	10 974	19.93
	18	2998	5.44
	31	1971	3.58
	33	3200	5.81
	35	667	1.21
	39	3454	6.27
	45	630	1.14
	51	3013	5.47
	52	11 282	20.49
	53	5513	10.01
	56	1419	2.58
	58	8007	14.54
	59	1276	2.32
	66	1749	3.18
	68	2813	5.11
LR-HPV	6	15 515	28.17
	11	2082	3.78
	42	536	0.97
	43	691	1.25
	44	571	1.04
	81	5008	9.09

### Age distribution in all positive samples

**Fig. 13.**
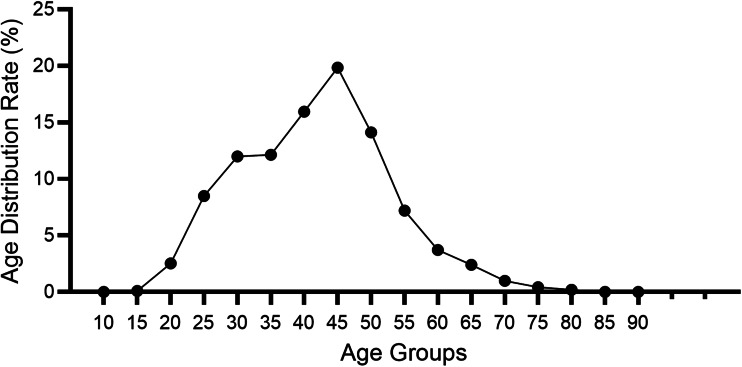
Age distribution rate in total population.

To better understand the age distribution of HPV infection, after dividing the subjects into 14 age groups (⩽15, 16–20, 21–25, 26–30, 31–35, 36–40, 41–45, 46–50, 51–55, 56–60, 61–65, 66–70, 71–75, ⩾76 years), the HPV infection prevalence of different regions in each age group was analysed (Table 2). It could be directly observed that HPV infection happened in all ages, and women aged 26–50 years accounted for a main part (Fig. 13). Elder women (>50) and young girls (⩽25) had a relatively lower HPV infection proportion. Besides, we conducted a *χ*^2^ test to determine whether the difference of age distribution was significant in different regions. The result showed that there was a significant difference among these groups (*χ*^2^ = 32.83, *P* < 0.001). We summarised the age distribution in three HPV infection patterns, including HR-HPV only, LR-HPV only, mixed HR-HPV and LR-HPV HPV infections, which showed that the pattern of HR-HPV only was the dominate infection one (Table 3).

**Table 2.** Age distribution of HPV infection in Western China

Age	Tibet	Chongqing	Guizhou	Shaanxi	Total
⩽15	2	21	23	5	51 (0.09%)
16–20	125	560	696	10	1391 (2.53%)
21–25	560	2579	1466	64	4669 (8.48%)
26–30	1139	4154	1157	147	6597 (11.98%)
31–35	1172	4698	640	171	6681 (12.13%)
36–40	1473	6568	565	174	8780 (15.94%)
41–45	2390	7657	657	220	10 924 (19.84%)
46–50	1557	5578	458	180	7773 (14.11%)
51–55	847	2706	260	146	3959 (7.19%)
56–60	334	1433	169	105	2041 (3.71%)
61–65	308	892	71	49	1320 (2.40%)
66–70	133	345	33	31	542 (0.98%)
71–75	73	145	13	8	239 (0.43%)
⩾76	37	53	11	3	104 (0.19%)
Total	10 150	37 389	6219	1313	55 071 (100%)

**Table 3.** Age distribution in different HPV infection patterns

	Type		
Age	HR only	LR only	Mixed
⩽15	32	13	6
16–20	852	313	226
21–25	3266	823	580
26–30	5047	993	557
31–35	5418	839	424
36–40	7187	968	625
41–45	8955	1275	694
46–50	6313	893	567
51–55	3137	450	372
56–60	1633	177	231
61–65	1043	107	170
66–70	428	46	68
71–75	196	12	31
⩾76	78	8	18
Total	43 585	6917	4569

### Age distribution of dominant types

**Fig. 14.**
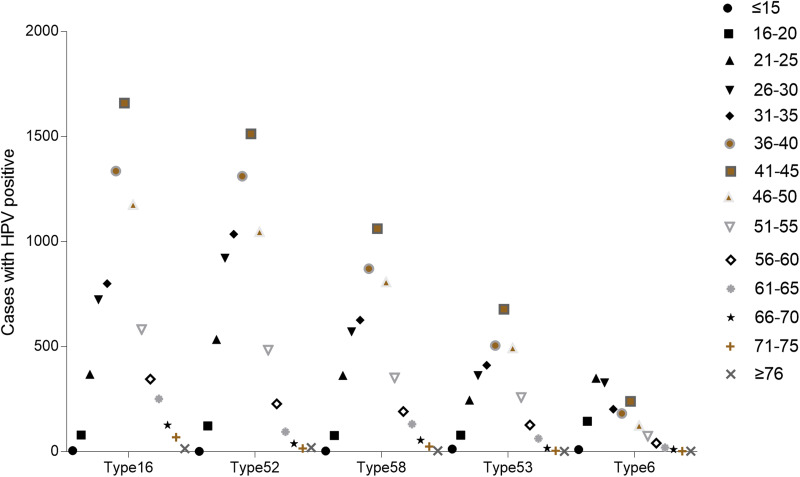
Age distribution of dominant HPV genotypes.

The most common five types of HPV were as follows on the basis of the above results: the HR-HPV genotype HPV 52, HPV 16, HPV 58, HPV 53 and LR-HPV 6. As for the five most prevalent genotypes, we decided to further explore their distribution characteristics in different age groups. Among type 52, 16, 58 and 53, the number of infection cases increased gradually and reached its highest point in the group aged 41–45 years old, while for groups aged over 41–45, the number of infection cases was reduced. Interestingly, in type 6, the most common infection age was found in women in 21–30 years old (Fig. 14). Women aged 36–40 years old and 46–50 years old also manifested an abundant number in these five genotypes HPVs. According to these results, we could come to the conclusion that women aged 36–50 years old account for the vast majority of these five dominant HPV infections.

## Discussion

A comprehensive estimation among women worldwide showed that HPV prevalence in Eastern Asia including China was significantly higher than that of both Southeastern Asia and South-central Asia, where HPV prevalence were 13.6%, 6.2%, 7.5%, respectively [5]. In addition, the prevalence of HPV in less developed countries (15.5%) was higher than that in more developed countries (10.0%), and Eastern Asia remains the most heavily burdened HPV region in Asia [5]. The number of HPV infection cases varies widely among Eastern Asian countries, as the most populous developing country, China faces a serious burden [16]. Moreover, it has been shown that Western China ranks the most in mortality rate and the second-most in cervical cancer incidence rate nationwide, thus the primary prevention of cervical cancer of Western China is particularly important [17].

In the present analysis, the overall HPV prevalence of 301 880 western Chinese women with normal cervical cytology was estimated to be at 18.24%, which was higher than the average global level, lower than that of many other countries, such as Eastern Africa, and Russia, and higher than that of Japan and India [5, 15]. A large comparable population-based study of HPV genotype prevalence nationwide showed a similar overall HPV infection of 21.07% with 120 772 samples from 37 cities in China tested [18], which was relatively higher than the overall HPV prevalence (18.24%) in this study. Regarding the HPV prevalence in different regional groups, this present survey also showed the high HPV prevalence: Tibet Autonomous Region (28.14%), Chongqing Municipality (18.59%), Guizhou Province (10.33%) and Shaanxi Province (29.09%). Compared with region-based data, the rates obtained in our study were different from those previously reported data from neighbouring regions, including Chongqing (26.20%) [19], Guizhou (16.95%) [20] and Yunnan (12.90%) [21] of Western China. The reported results of HPV prevalence vary from study to study as it is possibly caused by several variables, including the large Chinese population composition and territories. Together, the overall HPV-positive rate in the current study involving 301 880 cases was found to have increased slightly.

When stratified by HPV genotype, the most common HR-HPV types detected in our analysis were HPV52 (20.49%), which was inconsistent with the previous data generated by some Chinese population-specific investigations and some related studies reported that HPV 16 was identified as the most common HR HPV genotype [5, 18, 22–24]. The other three most prevalent HR/IR-HPV types were HPV16, 58 and 53, with frequencies of 19.93%, 14.52%, 10.01%, respectively. The characteristics of HPV distribution in our study were similar to a recent HPV study in Guizhou, China [20]. However, a recent study enrolling 37 722 females showed that the four most prevalent genotypes were HPV 16 (3.79%), HPV 52 (2.47%), HPV 58 (1.76%) and HPV 53 (1.35%) [17], which was different from our data. In addition, compared with a nationwide data of Chinese population-based research from 37 cities, except for HPV53 (not reported), the infection rates of HPV16 (4.82%), HPV52 (4.52%) and HPV58 (2.74%) were all lower than those in our study [18]. The knowledge of HPV prevalence and subtype distribution in different regions might facilitate the development of vaccination programme implementation. In this study, the HPV infection types and their proportions varied in different regions: the top three HPV genotypes were HPV 52, 16, 58 in Tibet Autonomous Region, HPV 16, 52, 58 in both Chongqing Municipality and Shaanxi Province and HPV 52, 16, 58 in Guizhou Province. Therefore, different regions showed diversity and had their respective proportions with respect to HPV genotypes. The differences in economic conditions, geographical cultural habits, migrations and other multiple factors might affect lifestyles among different populations, thus explaining the difference in the observed HPV prevalence.

Interestingly, among HR-HPV genotype infections, HPV53 type infection accounted for the top four in our study (10.01%). HPV53, a traditionally non-vaccine genotype, was recognised as a probable HR genotype and recently demonstrated to be associated with the putative potency of viral carcinogenicity (odds ratio, 3.92) [23, 25]. Moreover, the prevalence of the HPV53 genotype gradually elevated from 2011 to 2015 [23]. It was also reported as the fifth most common HPV type detected in Eastern Africa and Central and Northern America [5]. Thus, HPV prophylactic vaccines, including HPV53, may offer more sufficient protection for women in China.

In the present study, we found that the three most common LR-HPV types were HPV6 (28.17%), HPV81 (9.09%) and HPV11 (3.78%). A recent analysis involving 94 489 women from Eastern China conducted in 2019 has shown that the dominant LR-HPV genotypes were HPV81 and HPV6 [26]. Furthermore, a previous cross-sectional survey conducted in Arab women reported that HPV 81, 11 and 6 were the most commonly identified LR HPV genotypes in decreasing order [27], which were inconsistent with our research. The high HPV 6 prevalence in our study was unexpected. The reasons for this deviation of LR-HPV distribution are unclear and may be due to the cultural differences of the nationalities. Therefore, the HPV vaccine in China might also consider including the HPV 6 genotype.

When it comes to the HPV infection proportion of different age subgroups, the age distribution in this study showed that the middle age group (26–50 years) presented the highest HPV detection rate (75.53%, 71.82%, 62.75%, respectively) among HR HPV only, LR HPV only and mixed HPV genotype subgroups, with all subgroups indicating relatively lowest detection rate in elderly individuals (⩾76 years), similar to few recent studies in China [20, 28]. The most frequent age group of our HPV screening population was 25–50 years, which could explain the reason why the HPV types were distributed in this manner. Interestingly, we also concluded that the age distribution varied across different HPV genotypes in our study. As for the top four HR-HPV (type 52, 16, 58 and 53), the highest HPV detection rate was found in the group aged 41–45 years old, while the highest HPV detection rate in LR-HPV 6 infection in the group aged 21–30 years old group, which was different from a previous study concerning HPV genotyping results in China [22]. However, our results were consistent with the clinical phenomenon that increasingly frequent diagnoses of cervical cancer occurred in middle/old-aged Chinese women (40–64 years old). Therefore, it has become particularly important to disseminate information about cervical diseases as well as carry out HPV infection screening in China in line with the HPV distribution characteristics of each age group.

Among the patients infected with multiple subtypes, co-infections with two HPV types were the most common (76.04%) in our study, which was comparable to the regional results of Guangzhou, Sichuan and Macao in China from the previous reports [29–31]. Many epidemiologic studies found that infection with multiple HPV genotypes seemed to increase the risk of developing the tissue abnormalities or high-grade lesions that precede invasive cervical cancer considerably, because HPV types might interact synergistically, which might contribute to increasing the baseline risk observed with single-type infections [27, 32]. Similar results have been produced by other studies that confirmed its association with the development of the cervical carcinogenesis and increased the duration of the viral infection [33]. Therefore, it was suggested to consider diagnoses of co-infection with HPV into the prediction outcomes of HPV infections.

This is the first HPV distribution study with a large sample size in China. The principal strength of this study was that the large data enrolling 0.3 million women come from Western China, thus making it a representative of the general women population in China. One limitation of this study was sampling bias, because the most frequent age group of our HPV screening strategy was 25–50 years. Secondly, the women enrolled in this study attended to clinics for seeking medical advice based on routine gynaecological examination and HPV prevalence results, they always were accompanied by some clinical symptoms, which may lead to an over-reporting of HPV prevalence in this research. In addition, no clinical characters and cervical cytology results were collected as part of the study, resulting in the specific risk factors for the cervical cancer and the correlation between HPV genotype and cervical cancer or precancerous lesions were unable to be accurately examined. Furthermore, a follow-up study should be conducted to track changes in genotype, cervical pathology and cytology as there was a close relationship between cervical carcinoma and long-term persistent HR-HPV infections.

Thus, our results indicate that the preventative strategies including HPV vaccine-based popularisation and related educational campaigns should start in 25-year-old females. This information might provide valuable information for estimation of the potential clinical benefit of HPV-based screening in China.

## Conclusions

This study represents one of the most comprehensive studies of the prevalence and genotype distribution of different HPV types in China to date. We examined the epidemiology of HPV infection in China and confirmed the high overall HPV prevalence rate (18.24%) in all female subjects. Moreover, in addition to common genotype HPV52, 16 and 58, particular attention should be paid to the high prevalence of non-vaccine genotypes (e.g. HPV53, HPV6) in China. Therefore, the future next-generation HPV prophylactic vaccines in China should also consider to include more HPV types (e.g. HPV53, HPV6). Regarding the age-specific distribution of HPV, the highest HPV detection rate was found in age group 26–50 years old (detection rate = 74.00%). These results showed that the majority of HPV infections in China might be occurring in middle-aged women, which reminds us to attach great importance to middle-aged women in the prevention and control of HPV.

## Data Availability

The raw data are available on request by the editor of the publishing journal.

## Data availability statement

All data generated or analysed during this study are included in this published article.
